# Cloxacillin versus vancomycin for presumed late-onset sepsis in the Neonatal Intensive Care Unit and the impact upon outcome of coagulase negative staphylococcal bacteremia: a retrospective cohort study

**DOI:** 10.1186/1471-2431-5-49

**Published:** 2005-12-23

**Authors:** Sarah L Lawrence, Virginia Roth, Robert Slinger, Baldwin Toye, Isabelle Gaboury, Brigitte Lemyre

**Affiliations:** 1 Department of Obstetrics, Gynecology and Newborn Care, The Ottawa Hospital, Ottawa, Ontario, Canada; 2 Department of Microbiology, The Ottawa Hospital, Ottawa, Ontario, Canada; 3 Department of Pediatrics, Children's Hospital of Eastern Ontario, Ottawa, Ontario, Canada; 4 Chalmers Research Group, Children's Hospital of Eastern Ontario Research Institute, Ottawa, Ontario, Canada

## Abstract

**Background:**

Coagulase negative staphylococcus (CONS) is the main cause of late-onset sepsis in Neonatal Intensive Care Units (NICU). Although CONS rarely causes fulminant sepsis, vancomycin is frequently used as empiric therapy. Indiscriminate use of vancomycin has been linked to the emergence of vancomycin resistant organisms. The objective of this study was to compare duration of CONS sepsis and mortality before and after implementation of a policy of selective vancomycin use and compare use of vancomycin between the 2 time periods.

**Methods:**

A retrospective study was conducted of infants ≥4 days old, experiencing signs of sepsis with a first positive blood culture for CONS, during two 12-month periods. Late-onset sepsis was treated empirically with vancomycin and gentamicin during period 1, and cloxacillin and gentamicin during period 2. The confidence interval method was used to assess non-inferiority of the outcomes between the two study groups.

**Results:**

There were 45 episodes of CONS sepsis during period 1 and 37 during period 2. Duration of sepsis was similar between periods (hazard ratio of 1.00, 95%CI: 0.64, 1.57). One death during period 2 was possibly related to CONS sepsis versus none in period 1. Vancomycin was used in 97.8% of episodes in period 1 versus 81.1% of episodes in period 2.

**Conclusion:**

Although we failed to show non-inferiority of duration of sepsis in the cloxacillin and gentamicin group compared to the vancomycin and gentamicin group, duration of sepsis was clinically similar. Restricting vancomycin for confirmed cases of CONS sepsis resistant to oxacillin appears effective and safe, and significantly reduces vancomycin use in the NICU.

## Background

Coagulase negative staphylococcus (CONS) is the main cause of late-onset sepsis in neonatal intensive care units (NICU) [1]. Although fulminant late-onset sepsis (death occurring in less than 48 h) is usually due to gram-negative organisms, CONS may cause fulminant sepsis in about 1% of cases [1]. Increasing resistance of CONS to β-lactam antibiotics has prompted many NICUs to use vancomycin for initial treatment of presumed late-onset sepsis.

The use of vancomycin is an important risk factor for the emergence of vancomycin-resistant organisms, such as vancomycin-resistant enterococcus, and vancomycin-resistant *Staphylococcus aureus *[2-7]. These organisms can cause serious, life-threatening infections, but therapeutic options are limited. The possibility of developing an infection that cannot be treated with available antibiotics is a major public health concern. Restricting vancomycin use is considered important in preventing the development and spread of such organisms, and has been recommended by the Hospital Infection Control Practices Advisory Committee [8,9]. Critical care settings, such as the NICU, provide an ideal environment for the development and spread of resistant organisms due to the intensity of antimicrobial use, the close proximity of susceptible patients, and efficient vehicles of spread such as the hands of healthcare personnel. Therefore, finding effective alternatives to vancomycin for empiric therapy for sepsis in the NICU population would be an important achievement.

Several retrospective reviews suggest that restricting vancomycin use to confirmed cases of CONS sepsis is not associated with increased mortality or morbidity, even when oxacillin resistance rates are high [1,10-13]. However, these studies are limited by incomplete susceptibility data on the organism, and lack of a standard definition for duration of sepsis. Furthermore, the impact of removing the central venous catheter, if present, is not systematically accounted for, but can be a confounding factor in evaluating outcome [14-16].

A cluster of *S. aureus *sepsis in our NICU in early 2000 prompted a change in our initial therapy for presumed late-onset sepsis from vancomycin and gentamicin (non-restricted policy) to cloxacillin and gentamicin (restricted policy). In view of previous reports suggesting the probable safety of this practice, [1,10-13] and concerns about the emergence of antibiotic-resistant strains, we have continued to use cloxacillin and gentamicin as initial therapy, and to reserve vancomycin use for CONS sepsis with in-vitro resistance to oxacillin.

The primary aim of this study was to compare the duration of sepsis in infants who received cloxacillin with those who received vancomycin as empiric therapy for late-onset CONS sepsis. We hypothesized that restricting vancomycin use to oxacillin resistant organisms only, for late onset sepsis in the NICU would not be inferior to a non-restricted policy.

## Methods

### Study design

We conducted a retrospective chart review of infants with a positive blood culture for CONS and clinical signs of sepsis, treated with antibiotics during either of the two study periods. The study periods were defined as January 1, 1999 through December 31, 1999 (period 1) and April 1, 2000 through March 31, 2001 (period 2). Initial therapy for sepsis consisted of intravenous vancomycin (15–20 mg/kg/dose) plus gentamicin (2.5 mg/kg/dose) during period 1, and intravenous cloxacillin (50 mg/kg/dose) plus gentamicin (2.5 mg/kg/dose) during period 2. The new protocol for empiric therapy was implemented between January and March 2000, and data from this period are not included as practices were inconsistent. During period 2, vancomycin could be used instead of cloxacillin and gentamicin only if the organism was oxacillin-resistant. The policy included stopping gentamicin once the CONS susceptibility profile was available, and the infant had clinically improved. The susceptibility profile was typically available at 48 to 72 hours. Patients routinely had pre- and post-drug levels drawn with the 3^rd ^dose of vancomycin and gentamicin. Drug dose was adjusted based on these results to ensure levels remained therapeutic.

### Study population

All infants 4 days of age or older (day of birth counting as day 1) diagnosed with a first CONS sepsis in the NICU of The Ottawa Hospital, General Campus (Ontario, Canada) during the two study periods were eligible for this study. CONS sepsis was defined as one positive blood culture for CONS (within 48 hours of incubation) plus one or more of the following signs of infection: lethargy, increased frequency of apneic spells over baseline, temperature instability of more than 1 degree Celsius over 24 h, need for intubation or increased ventilatory support, or poor perfusion requiring fluid boluses or inotropic support. Episodes were excluded as contaminants if they did not meet these clinical criteria for CONS sepsis, or if blood culture isolated either a second non-CONS organism or a second CONS strain. Repeat episodes of CONS sepsis were excluded.

The NICU of The Ottawa Hospital General Campus is a regional tertiary perinatal care center. The hospital serves a population of one million people, with 18 000 live births per year. The NICU admits approximately 350 infants annually, 150 (43%) of whom weigh less than 1500 grams at birth. The rate of methicillin-resistant *Staphylococcus aureus *(MRSA) in this facility is approximately 17 per 100 000 patient days. In the past 10 years, MRSA has been isolated from only two NICU infants. In both instances the isolate represented colonization, rather than infection, and the incidences were not epidemiologically linked. There have been no documented isolates of vancomycin-resistant enterococcus, or vancomycin-resistant *Staphylococcus aureus *in our NICU.

### Laboratory methods

A single peripheral blood culture was obtained from each infant in whom late-onset sepsis was suspected. The site of venipuncture was prepared by cleansing the skin with an antiseptic swab impregnated with 0.5% w/v chlorhexidine gluconate and 70% v/v isopropyl alcohol in a circular motion, beginning at the site and working outward for a 3–5 cm diameter. The site was allowed to dry for 60 seconds and then 0.5 ml of blood was drawn using a 25G butterfly needle. The blood was inserted into a blood culture vial (BacT/ALERT PF Pediatric bottle, BioMérieux Inc., Durham, NC, USA). There was no standard protocol for repeat of blood cultures. If the baby remained clinically unwell, or if antibiotics were switched due to culture insensitivity, cultures were routinely repeated prior to changing or adding antibiotics. Cultures of catheter tips, if removed, were not routinely obtained.

Coagulase-negative staphylococci were identified using standard microbiological methods but were not further speciated. Oxacillin and gentamicin susceptibilities were determined by the disk diffusion method following the National Committee for Clinical Laboratory Standards. The interpretive criteria used for resistance have been shown to correlate highly with the presence of the *mecA *gene, which is the genetic determinant for oxacillin resistance in staphylococci [17].

### Outcomes

The primary outcome for the study was duration of sepsis, defined as either the length of time from the first positive blood culture for CONS until the first documented negative blood culture (in days), or the time to clinical recovery (in days). Because we cannot be sure of either the exact time of onset of sepsis, or of clinical recovery, duration of sepsis can only be considered accurate to within one day. If there was apparent clinical recovery, but persistently positive blood cultures, duration of sepsis was defined as time to negative blood culture. Clinical recovery was defined as return to prior clinical status including resolution of lethargy and temperature instability, extubation if previously non-ventilated, or return to previous level of ventilatory support, the end of inotropic support, and the same average daily number and quality of apneic spells as before the onset of sepsis.

Secondary outcomes were mortality rate, within 14 days of the first positive blood culture for CONS, duration of sepsis with and without a central line, and vancomycin use during the two study periods. Gentamicin susceptibility of the CONS strains implicated in each sepsis episode was determined to account for the potential impact of concurrent gentamicin use on the primary outcome.

The Ottawa Hospital Research Ethics Committee approved this study.

### Statistical analysis

Summary statistics were expressed as frequencies, or as the mean ± standard deviation, median and range. Baseline characteristics were compared between the two study groups using Student's t-test and Fisher's exact test. Birth weight, gestational age and age at onset of sepsis were not normally distributed. Therefore, statistical comparisons have been run on the log-transformed data. Length of time between sepsis and antibiotics was compared using a log-rank test. Use of vancomycin was compared using Fisher's exact test.

Unlike traditional hypothesis testing approaches, where the research hypothesis is that two interventions differ, in a non-inferiority study the research hypothesis is that one intervention is not clinically inferior to the other. This requires the prior specification of a difference large enough to clinically favour the standard intervention over the study intervention. The study intervention is declared non-inferior to the standard intervention when the confidence interval contrasting them only covers values smaller than this prespecified amount [18]. Differences in duration of sepsis, adjusted for lethargy, were measured using the hazard ratio and its 95% confidence interval (CI), estimated using Cox regression. Assuming that duration of sepsis with the 1999 protocol (non-restricted vancomycin use) was about 3 days [1,12], a clinically inferior duration of sepsis was taken to be 4 days, corresponding to a margin of inferiority on the hazard ratio scale of 1.33. For the secondary outcome, difference in proportion of mortality was computed with its 95% CI estimated using the Wilson score method [19]. The margin of non-inferiority was taken to be a 1% difference in mortality.

## Results

There were 54 positive CONS blood cultures in period 1 (1999) and 43 positive blood cultures in period 2 (2000–2001). Six of these episodes (three in each period) did not meet the clinical definition for CONS sepsis (two infants had concomitant fungal or gram negative sepsis, two did not receive antibiotics and two had no clinical sign of sepsis) and were excluded as likely contaminants. Six cultures in period one and three in period two were excluded because they were second episodes of CONS sepsis. Baseline characteristics of the excluded infants/episodes were comparable to those of the included ones. Baseline characteristics of the remaining 45 infants in period 1 and 37 infants in period 2 are presented in Table 1.

**Table 1:** Characteristics of Infants Treated for Coagulase-Negative *Staphylococcus*

	**Period 1: 1999 (No vancomycin restriction) (n = 45)**	**Period 2: 2000–2001 (Vancomycin restriction) (n = 37)**	**Difference (95% CI)**	**p-value**
**Baseline characteristics, median (range)**				
Birth weight, grams	890 (550, 1750)	966 (628, 3417)	39 (-69, 148)	0.269
Gestational age, weeks	27 (24, 31)	27 (24, 41)	0.0 (-1.0, 1.0)	0.337
Age at onset of sepsis, days	13 (4, 74)	10 (5, 23)	-2 (-4, 1)	0.149
**Characteristics at onset of sepsis, n (%)**				
Presence of a central venous line	18 (40.0)	21 (56.8)	16.8 (-4.8, 36.3)	0.183
Central venous line removal *	10/18 (55.6)	10/21 (47.6)	7.9 (-35.6, 21.7)	0.751
Receiving total parenteral nutrition	41 (91.1)	36 (97.3)	6.2 (-6.2, 18.2)	0.372
Prior antibiotics	43 (95.6)	33 (89.2)	-6.4 (-20.6, 5.9)	0.402
Mean time between sepsis and prior antibiotics, days (sd)	6.4 (4.4)	4.7 (3.4)	-1.7 (-3.5, 5.8)	0.053
**Clinical signs of sepsis, n (%)**				
Lethargy	41(91.1)	25 (67.6)	-23.5 (-40.5, -6.1)	0.011
Increased frequency of apneic spells	35 (77.8)	23 (62.1)	-15.6 (-34.4, 4.1)	0.148
Temperature instability	23 (51.1)	17 (45.9)	-5.2 (-25.6, 15.9)	0.664
Need for increased ventilatory support	9/12 (75.0)	15/16 (93.7)	18.8 (-8.6, 47.4)	0.285
Need for intubation in infants not on any ventilatory support	20/33 (60.6)	14/21 (66.7)	6.1 (-19.8, 29.4)	0.775
Poor perfusion requiring fluid boluses or inotropic support	15 (33.3)	11 (29.7)	-3.6 (-22.7, 16.4)	0.814

**Isolate resistant, n (%)**				

Cloxacillin	34 (75.5)	29 (78.4)	2.8 (-15.8, 20.4)	0.799
Gentamicin	30 (66.7)	26 (72.2)	3.6 (-16.4, 22.7)	0.635
Both cloxacillin and gentamicin	23 (51.1)	26 (72.2)	21.1 (-0.03, 39.6)	0.113

* Percentage based on infants with a central venous line

In period 1, 44 of the 45 episodes of CONS sepsis were treated empirically with vancomycin and gentamicin. In 11 of these 44 episodes, antibiotics were switched to cloxacillin when susceptibilities were available. Two of the 44 infants were given a dose of ampicillin before vancomycin was initiated. In the 45^th ^episode, the infant received ceftazidime and gentamicin, which was changed to cloxacillin after identification of CONS. In period 2, 35 of the 37 episodes were treated empirically with cloxacillin and gentamicin. In 28 of these 35 episodes, antibiotics were changed to vancomycin when the CONS isolate was determined to be resistant to oxacillin. In two episodes, cloxacillin was continued despite resistance as the infant had clinically improved. These two isolates were also resistant to gentamicin, however the central line was removed in one infant during the sepsis. Two episodes in period 2 were treated empirically with vancomycin. In one of these episodes the antibiotic coverage was switched to cloxacillin when the organism was shown to be susceptible to oxacillin.

Comparisons of the main outcomes are shown in Table 2. The mean duration of sepsis was comparable between study periods, 3.6 days (Range: 1 to 10 days) versus 3.4 days (Range: 1 to 12 days); although non-inferiority of a restricted vancomycin policy could not be demonstrated potentially due to insufficient sample size. In surviving infants, duration of sepsis was documented as either the length of time from the first positive blood culture for CONS until the first documented negative blood culture (14 episodes in period 1 and 16 episodes in period 2) or as the length of time to return to previous clinical status (31 episodes in period 1 and 17 episodes in period 2).

**Table 2:** Outcome of Infants Treated for Coagulase-Negative *Staphylococcus*

**Variables**	**Period 1 1999 (No vancomycin restriction) (n = 45)**	**Period 2 2000–2001 (Vancomycin restriction) (n = 37)**	**Hazard ratio (95 %CI)**	**Difference of % (95% CI)**
Mean duration of sepsis, days (sd)	3.6 (2.5)	3.4 (3.0)	1.2 (0.8, 2.0)*	N/A
Deaths due to CONS sepsis, n (%)	0 (0.0)	1 (2.7)	N/A	-2.7 (-13.8, 5.5)
14 day mortality, n (%)	0 (0.0)	4 (10.8)	N/A	-10.8 (-24.7, 0.6)

* Hazard ratio based on the Cox regression model

No infants died during the 14 days after positive CONS blood culture during period 1, and four infants died during this time in period 2. Three of these deaths were considered unrelated to sepsis. One infant was critically ill with severe chronic lung disease and pulmonary interstitial emphysema when she developed CONS sepsis. A decision was made to withdraw therapy based on poor lung function. The other two infants had documented negative blood cultures for CONS several days before their deaths, which were felt to be due to underlying comorbidities. One of these two infants developed an *Escherichia coli *sepsis 5 days into treatment for CONS, while treated with vancomycin. The second infant died after therapy was withdrawn because of worsening pulmonary interstitial emphysema and lung blebs, recurrent pneumothorax and grade II and IV intraventricular hemorrhages. One death was considered possibly related to CONS sepsis. The patient was 25 weeks gestation, born weighing 831 g, who became sick at 2 weeks of age, with apneic spells, temperature instability, poor perfusion and increased ventilator requirements. He was started on cloxacillin and gentamicin. The CONS strain in this case was resistant to both oxacillin and gentamicin. Because of clinical deterioration, vancomycin was added to therapy before the susceptibility results were available, however the infant died the same day. An autopsy was not performed to confirm the clinical impression of death due to CONS sepsis.

A total of 39 infants in the two periods had central venous lines at the onset of sepsis (Table 1). When adjusted for therapy protocol, and lethargy, duration of sepsis with a line present was statistically longer than when no line was present, 4.6 (3.3 sd) versus 2.6 (1.7 sd) days; p = 0.019.

Use of vancomycin for CONS sepsis decreased from 44 out of 45 episodes (97.8%) in period 1 to 30 out of 37 episodes (81.1%) in period 2 (p = 0.020). In total, in period 1 790 doses of vancomycin were given for CONS sepsis, compared to 347 doses in period 2. Including CONS sepsis episodes, there were a total of 96 episodes of presumed late-onset sepsis during period 1 and 102 episodes during period 2. The majority of these episodes were culture negative and antibiotics were stopped. Included in the 198 episodes of presumed late-onset sepsis in both periods were 17 episodes of sepsis with another organism {period 1: *Staphylococcus aureus *(1), *Escherichia coli *(2), *Enterobacter cloacae *(2), *Pseudomonas aeruginosa *(1), *Enterococcus faecalis *(1); period 2: *Staphylococcus aureus *(3), *Escherichia coli *(2), *Enterobacter cloacae *(1), *Candida albicans *(3), Group A Streptococcus (1)}. Vancomycin was used in a total of 82/96 (85.4%) episodes (1042 doses) in period 1 compared to 42/102 (41.2%) episodes (409 doses) in period 2 (p < 0.001).

The majority of CONS strains were resistant to oxacillin, gentamicin or both (Table 1). One isolate, in period 2, failed to grow on susceptibility testing media for gentamicin. In period 2, 29 isolates were resistant to oxacillin. In 27 of these cases, antibiotics were switched to vancomycin, however 14 (52%) of these 27 infants had clinically improved from their sepsis before the start of vancomycin: 10 had returned to their baseline clinical status, 2 had a negative blood culture and 2 had both before initiation of vancomycin. Two of these 14 CONS isolates were sensitive to gentamicin, and another was taken from an infant who had his central venous line removed after the onset of sepsis but before the vancomycin was given. The remaining 11 infants recovered with cloxacillin and gentamicin while sensitivity testing indicated in-vitro resistance to both oxacillin and gentamicin.

## Discussion

This is one of the largest studies aimed primarily at comparing outcomes in neonates with CONS sepsis. Although non-inferiority of a restricted vancomycin policy could not be demonstrated (potentially due to insufficient sample size), the mean duration of sepsis among infants treated with empiric cloxacillin and gentamicin was comparable to that among infants treated with empiric vancomycin. Mortality was low in both groups of infants in this study. There was one death in an infant treated with empiric cloxacillin and gentamicin that may have been linked to CONS sepsis; however, insufficient clinical information was available to establish causality.

These results are consistent with previously published studies [1,10,11]. Karlowicz et al [1] reviewed all cases of late-onset sepsis, including 277 episodes of CONS sepsis, over a 10-year period in one institution. The number of episodes, duration and mortality rates were similar whether vancomycin and cefotaxime or cloxacillin and gentamicin were used empirically. However, no information regarding the presence or removal of a central venous line, sensitivity data or details about baseline characteristics of included infants were available, thus limiting generalizability. Matrai-Kovalskis et al [10] reviewed 127 episodes of positive blood cultures for CONS of which only 22 were considered true sepsis. Clinical signs were not part of the definition of sepsis. These episodes were compared to 105 episodes selected randomly from a sample of 210 considered contaminants. Infants in both groups were treated with ceftazidime+/- ampicillin or cloxacillin; vancomycin was reserved for confirmed cases of CONS. There was no difference in rates of recovery between the groups, although three infants in the "contaminant" group died and several received a full antibiotic course. Krediet et al [11] reviewed 66 cases of CONS sepsis, treated with cephalothin alone, vancomycin alone or cephalothin then vancomycin once resistance to oxacillin was confirmed. Mortality and duration of sepsis were similar between the 3 groups, even though CONS was resistant to oxacillin in 85% of cases. None of these studies reported differences in death rates between groups; however, they were not powered for death as an outcome. Although these data are reassuring, the limited sample size in the present study does not permit the demonstration of non-inferiority in mortality between the two patient groups.

Most CONS isolates recovered from infants in this study were resistant to oxacillin and/or gentamicin. Nevertheless, similar to Krediet et al's findings [11], we documented clinical improvement in more than half of the infants before the initiation of vancomycin, even in the face of oxacillin resistance. One possible explanation is that the CONS isolate represented a contaminant rather than true bacteremia. Although all study patients met the clinical criteria for sepsis, it is possible that their deterioration was due to other causes. Secondly, the susceptibility results based on phenotypic testing may indicate resistance despite the absence of the *mecA *gene for less commonly encountered species of CONS (e.g. *S. lugdenensis*). However, these species are rarely encountered in our hospital including the NICU, and we have previously shown that the disk diffusion method for oxacillin susceptibility testing correlates highly with the presence or absence of the *mecA *gene [17]. Third, removal of the central line may lead to clearance of CONS bacteremia, even in the absence of antibiotic use. Finally, it may be that the addition of gentamicin to cloxacillin results in an in vitro synergistic antibiotic effect on staphylococci [20]. However, a literature search failed to reveal any published studies demonstrating such a clinical benefit when patients are treated with combination therapy for infections due to CONS isolates that are resistant to oxacillin in vitro.

This study has several limitations. Although this is one of the largest studies evaluating outcomes in CONS sepsis in neonates, we undertook this study as part of a quality assurance process to compare similar time periods rather than a pre-determined number of sepsis episodes. Thus, the sample size limits the strength of our findings. Second, even with a clear definition of recovery from sepsis, it is difficult to be certain that sepsis had resolved. Not all infants had a documented repeat blood culture to confirm clearance of the organism, therefore duration of sepsis was recorded in days, rather than hours, which would have allowed for more precise comparison. We attempted to minimize this bias by having a single investigator responsible for assigning duration of sepsis based on a pre-specified definition for all infants. Third, the use of historical controls introduces the potential for bias, as there may be unaccounted for differences between infants in the two periods that could influence outcome. We chose comparison periods as close as possible in time to try to minimize this bias and baseline characteristics of included infants were similar between periods. A fourth potential limitation is the use of a single blood culture for infants exhibiting signs of sepsis. CONS is a frequent contaminant and Struthers et al [21] estimated that an additional 31% of infants were diagnosed as having CONS sepsis when diagnosis was based on only one blood culture as compared to two blood cultures. But, more recently, Sarkar et al [22] found no advantage in drawing two blood cultures for diagnosing sepsis in 216 neonates. We tried to limit the number of contaminants by careful preparation of the skin prior to drawing blood culture, taking the culture from peripheral sites rather than central lines, and considering the CONS to be a contaminant if the isolate was recovered after 48 hours or more of incubation. Furthermore, we included only infants with simultaneous clinical signs of sepsis. Using these criteria, six instances of CONS positive blood cultures were excluded from the study as likely contaminants. A final potential bias is the presence of a central venous line. Our data suggests a longer duration of sepsis when a central line was present, however there is no policy in our unit for line removal with sepsis. The presence of foreign material in the bloodstream allows for the formation of biofilm-incorporating microorganisms, which are very difficult to treat [23] and can prolong the duration of sepsis [14-16].

Reserving vancomycin for the treatment of sepsis caused by organisms confirmed to be oxacillin resistant is an important measure to control the spread of vancomycin resistant organisms. The results of this study suggest that empiric vancomycin use for late-onset neonatal sepsis may not be warranted in facilities with a low incidence of MRSA infections. Although we failed to demonstrate that a restricted vancomycin policy is not inferior in terms of duration of sepsis or mortality from CONS sepsis as compared to a non-restricted policy, we observed similar duration of sepsis (considering that duration estimate can only be accurate to within 1 day), and mortality was low in both groups. Given the high incidence but low mortality and morbidity from CONS sepsis in the NICU, vancomycin could be reserved for episodes of late-onset sepsis in which the implicated organism is confirmed to be oxacillin resistant. A policy change for empiric treatment of late-onset sepsis led to an important reduction in vancomycin use in our NICU. A prospective randomized controlled trial, powered to adequately assess similar morbidity and mortality rates is warranted to confirm these findings. Rubin et al [24] recommend that clinical practice guidelines should be developed in NICUs to ensure adequate blood sampling to decrease contamination, obtaining 2 blood cultures and possibly using adjunctive tests and information to help differentiate contaminants from pathogens. These guidelines would assist greatly in conducting such a trial. As well, routinely obtaining repeat blood cultures to document clearance of the organism would be helpful in defining length of sepsis and a central line removal policy would be beneficial in limiting bias between study groups.

## Competing interests

The author(s) declare that they have no competing interests.

## Authors' contributions

SL contributed to the design of the study, data gathering, and drafting of the manuscript. VR, RS and BT contributed to the design of the study, and the revision of the manuscript. IG participated in the design of the study and performed the statistical analysis. BL conceived the study, participated in design, interpretation of data, and drafting of the manuscript. All authors read and approved the final manuscript.

## Pre-publication history

The pre-publication history for this paper can be accessed here:



## References

[B1] Karlowicz MG, Buescher ES, Surka AE (1997). Fulminant late-onset sepsis in a neonatal intensive care unit, 1988-and the impact of avoiding empiric vancomycin therapy. Pediatrics.

[B2] Bonten MJ, Willems R, Weinstein RA (2001). Vancomycin-resistant enterococci: why are they here, and where do they come from?. Lancet Infect Dis.

[B3] Martone WJ (1998). Spread of Vancomycin-resistant enterococci: why did it happen in the United States?. Infect Control Hosp Epidemiol.

[B4] Garrett DO, Jochimsen E, Murfitt K, Hill B, McAllister S, Nelson P, Spera RV, Sall RK, Tenover FC, Johnston J, Zimmer B, Jarvis WR (1999). The emergence of decreased susceptibility to vancomycin in Staphylococcus Epidermidis. Infect Control Hosp Epidemiol.

[B5] Veach LA, Pfaller MA, Barrett M, Koontz FP, Wenzel RP (1990). Vancomycin resistance in Staphylococcus Haemolyticus causing colonization and bloodstream infection. J Clin Microbiol.

[B6] (2002). Public health dispatch: vancomycin-resistant Staphylococcus Aureus. Morbidity and mortality weekly report.

[B7] (2002). Staphylococcus Aureus resistant to vancomycin. Morbidity and mortality weekly report.

[B8] (1997). Interim guidelines for prevention and control of staphylococcal infection associated with reduced susceptibility to vancomycin. Morbidity and mortality weekly report.

[B9] Hospital Infection Control Practices Advisory Committee (1995). Recommendations for preventing the spread of vancomycin resistance. Infect Control Hosp Epidemiol.

[B10] Matrai-Kovalskis Y, Greenberg D, Shinwell ES, Fraser D, Dagan R (1998). Positive blood cultures for coagulase-negative staphylococci in neonates: does highly selective vancomycin usage affect outcome?. Infection.

[B11] Krediet TG, Jones ME, Gerards LJ, Fleer A (1999). Clinical outcome of cephalothin versus vancomycin therapy in the treatment of coagulase-negative staphylococcal septicemia in neonates: relation to methicillin resistance and mec A gene carriage of blood isolates. Pediatrics.

[B12] Lam JPH, Mitchell SJ, Gore C (2001). Selective use of vancomycin for empirical treatment of neonatal sepsis: effects on clinical outcome and glycopeptide usage. Arch Dis Child.

[B13] Allen TR, da Silva OP (2003). Choice of antibiotics in late neonatal sepsis in the extremely low birth weight infant. Can J Infect Dis Med Microbiol.

[B14] Furigay PJ, Karlowicz MG, Croitoru DP, Buescher ES (2000). Coagulase-negative staphylococcal bacteremia in neonates: Can it be treated successfully with vancomycin without removing central venous catheters?. Pediatr Res.

[B15] Karlowicz MG, Furigay PJ, Croitoru DP, Buescher ES (2002). Central venous catheter removal versus in situ treatment in neonates with coagulase-negative staphylococcal bacteremia. Pediatr Infect Dis J.

[B16] Benjamin DK, Miller W, Garges H, Benjamin DK, McKinney RE, Cotton M, Fisher RG, Alexander KAl (2001). Bacteremia, central catheters, and neonates: when to pull the line. Pediatrics.

[B17] Woods W, Ramotar K, Lem P, Toye B (2002). Oxacillin susceptibility testing of coagulase-negative staphylococci using the disk diffusion method and the Vitek GPS-105 card. Diag Microbiol Infect Dis.

[B18] Jones B, Jarvis P, Lewis JA, Ebbutt AF (1996). Trials to assess equivalence: the importance of rigorous methods. BMJ.

[B19] Newcombe RG (1998). Interval estimation for the difference between independent proportions: comparison of eleven methods. Stat Med.

[B20] Watanakunakorn C, Glotzbecker C (1974). Enhancement of the effects of anti-Staphylococcal antibiotics by aminoglycosides. Antimicrob Agents Chemother.

[B21] Struthers S, Underhill H, Albersheim S, Greenberg D, Dobson S (2002). A comparison of two versus one blood culture in the diagnosis and treatment of coagulase-negative staphylococcus in the Neonatal intensive care unit. J Perinatol.

[B22] Sarkar S, Bhagat I, Mele P, Herbert C, Wiswell TE, Spitzer AR (2003). A prospective trial of multiple site blood cultures in the initial evaluation of neonatal sepsis [abstract]. Pediatr Res.

[B23] Donlan RM (2001). Biofilms and Device-Associated Infections. Emerging infectious disease.

[B24] Rubin LG, Sanchez PJ, Siegel J, Levine G, Saiman L, Jarvis WR (2002). Evaluation and treatment of neonates with suspected late-onset sepsis: a survey of neonatologists' practices. Pediatrics.

